# Beyond technical access in digital eldercare: how ethical lag shapes stratified responsiveness to institutional welfare in rural China

**DOI:** 10.1186/s12877-026-07768-1

**Published:** 2026-06-10

**Authors:** Yuhe Liu, Ying Wang, Mengxin Xu, Haoyu Dong, Yue Zhang

**Affiliations:** 1 School of Public Policy and Administration, Chongqing University, Chongqing, China; 2 Faculty of Education, University of Macau, Macao, China

**Keywords:** Digital Eldercare, Ethical Lag, Care Acceptability, Behavioral Intention, Stratified Responsiveness, Rural China

## Abstract

**Background:**

Digital elder care services have been widely promoted to address population ageing, yet their uptake among rural older adults remains limited even when access and affordability are ensured. Existing studies largely attribute this gap to digital literacy or technical barriers, while insufficient attention has been paid to the moral and ethical meanings through which older adults interpret institutional care.

**Methods:**

This study adopted a mixed-methods design in Shapingba District, Chongqing, China. Quantitative data were collected from 400 rural older adults aged 60 and above and analysed using structural equation modelling. In-depth semi-structured interviews with 15 outliers from the quantitative phase were conducted to explore moral reasoning, care expectations, and lived experiences with digital services.

**Results:**

Quantitative results showed that Care Acceptability was the most consistent direct predictor of behavioral intention. By contrast, Privacy and Data Protection and Dignity in Care exerted weaker direct effects and were more likely to influence behavioral intention indirectly through perceived ease of use and perceived usefulness. Qualitative findings further identified three response patterns: adaptive translation, ethical dissonance, and translation failure. These patterns reflect an ethical lag, whereby localized care expectations adapt more slowly than technological reforms, leading to stratified engagement among rural older adults.

**Conclusions:**

The findings suggest that limited uptake of digital elder care is closely tied to moral legitimacy and ethical recognition. Digital care services are more likely to be accepted when perceived as a supportive extension of, rather than a replacement for, family care responsibilities. Addressing ethical lag through culturally resonant service design and trusted community mediation may help reduce inequalities in engagement and improve the effectiveness of ageing-related service provision.

**Supplementary Information:**

The online version contains supplementary material available at 10.1186/s12877-026-07768-1.

## Introduction

The global expansion of digital welfare systems has intensified scholarly concern with the relationship between technological innovation and institutional legitimacy in public service delivery [28, 36]. As governments increasingly adopt digital infrastructures to manage long-term care for aging populations, a range of challenges has emerged concerning inclusion, trust, and value alignment [10]. Whether through algorithmic assessments in eldercare platforms or automated allocation in community services, digital technologies often struggle to accommodate the normative expectations embedded in caregiving cultures [26, 38].

In East Asian societies where family-based caregiving has long served as a central pillar of social organization, this tension becomes particularly visible. In China, the state has promoted a wide range of digital eldercare initiatives, such as *smart community platforms*, *Internet plus nursing services,* and *AI-based monitoring programs *[33, 34]. These efforts aim to address the demands of population aging while building a more efficient, modernized social service infrastructure. However, in rural areas, these services frequently encounter forms of quiet resistance. Older adults often exhibit hesitation or avoidance in using institutional digital care services, even when they are low-cost, subsidized, or readily available.

Existing explanations for this limited uptake tend to fall into two main categories. One line of research emphasizes instrumental or functional concerns, such as digital literacy, technological accessibility, and perceived ease of use [16, 19]. Another stream highlights emotional and relational aspects, including service satisfaction, perceived dignity, and relational trust in family or community ties [12, 22]. While both perspectives contribute valuable insights, they often interpret elder users’ choices as individualized behavioral outcomes, neglecting the deeper moral structures and social contexts in which care decisions are made.

In rural China, care is not merely a matter of service provision but a reflection of moral commitments situated within enduring kinship norms and intergenerational obligations. The introduction of institutionalized digital care arrangements challenges traditional understandings of who should care, how care is recognized, and what it means to “be filial”. Many rural elders perceive publicly provided digital care not as neutral assistance but as a potential disruption of family responsibilities. As such, the question of whether one accepts or avoids institutional support often invokes more than concerns of convenience; it becomes a judgment about moral legitimacy and role displacement within the family structure.

This paper moves beyond individual acceptance models to examine the institutional and ethical structures that shape rural elders’ responsiveness to digital eldercare. It asks:

- How do elderly individuals in rural China assess the legitimacy of digital care arrangements?
- How do intergenerational norms and ethical expectations influence their willingness to engage with publicly provided digital services?

In answering these questions, the study brings ethical reasoning and familial normativity into dialogue with public management frameworks, highlighting the ways in which institutional responsiveness is shaped not only by system design or service quality but also by the compatibility between policy instruments and local moral ecologies.

## Literature review and research hypotheses

### Family ethics as the moral foundation of elder care decisions

In rural China, elder care is not primarily determined by institutional availability or policy incentives but is deeply embedded in family ethics. Drawing from structural-functionalist theory, the family is viewed not only as a material support unit but also as a normative institution that allocates caregiving responsibilities through culturally encoded role expectations [4, 7]. Within this framework, elder care is morally justified only when performed by close kin within the private domestic sphere [15, 21]. The introduction of digital surveillance and health monitoring potentially disrupts these lineage and inheritance norms by manifesting as an external intrusion into the private family domain. Therefore, *Privacy and Data Protection (PDP)* concerns may serve as a proxy for assessing whether the symbolic moral boundaries of the private domestic sphere are maintained in the face of institutional intervention.

Beyond role legitimacy, the emotional experience quality of caregiving interactions matters deeply. Care is not perceived as meaningful unless it evokes emotional familiarity, dignity, and mutual recognition—qualities historically reproduced in cohabitation, shared rituals, and embodied intimacy [1, 30]. When digital equipment replaces or mediates in-person care, it risks stripping away the warmth of filial piety, potentially leading to a sense of emotional neglect or a loss of individual agency. Thus, *Dignity in Care (DC)* serves as a critical ethical barometer for older adults to evaluate whether digital elder care honors their emotional status and personhood.

Moreover, elder care in rural villages is publicly evaluated within communal moral economies. Fulfilling filial duties enhances familial reputation and moral standing, while outsourcing care risks reputational loss [11, 39]. However, as digital welfare becomes a new institutional norm, the moral stigma of outsourcing may be mitigated if there is a shift in communal consensus regarding the legitimacy of smart care. *Care Acceptability (CA)* thus reflects the alignment between these evolving social expectations and the elderly’s own moral endorsement of technological alternatives.

These dimensions serve as a foundational lens through which older adults interpret digital elder care. If such services lack alignment with these familial and communal ethics, they are likely to be judged as morally deficient regardless of their objective technical quality [2, 31]. Consequently, the adoption of digital care is a moral decision where ethical legitimacy, s assessed through perceptions of PDP, DC, and CA, appears to provide a normative basis for navigating potential cultural resistance. Based on this logic, the paper propose:

H1a: Privacy and Data Protection positively influences the behavioral intention of rural older adults to adopt digital care services.

*H1b: Dignity in Care positively influences the behavioral intention of rural older adults to adopt digital care services.*

*H1c: Care Acceptability positively influences the behavioral intention of rural older adults to adopt digital care services.*

### Social ethics as the institutional translation mechanism of family care norms

As China’s welfare institutions expand and elder care transitions toward digitalized delivery, formal services increasingly operate beyond the bounds of kinship-based moral orders [20, 24]. [In this context, institutional acceptance depends less on procedural efficiency than on the capacity to reconstruct moral legitimacy in ways that resonate with deeply embedded family ethics. This process entails the institutional translation of familial moral convictions into culturally intelligible frameworks of public care.

The first requirement is the construction of ethical role identity. In family based care, legitimacy is self-evident, rooted in blood ties and intergenerational obligation. In contrast, institutional actors must actively establish their moral authority. As Giddens [13] suggests, modern institutions must render their moral purpose visible to sustain trust in the absence of traditional legitimacy. Similarly, Boltanski [5] argues that social action requires justification within shared regimes of worth. Without recognizable ethical signals, institutional care is unlikely to be perceived as morally appropriate.

Second, institutions must achieve emotional resonance. Moral legitimacy is not conferred solely through functional adequacy but through affective recognition [6]. Emphasize that institutions must align with individuals’ moral worldswhere concepts like empathy, dignity, and presence are central. Emotionally sterile or proceduralized care systems often alienate rural elders, who equate moral worth with interpersonal warmth and symbolic attentiveness [32].

Third, institutional care must obtain public moral endorsement. In rural “acquaintance societies” [11], elder care is evaluated not privately but within communal moral economies. Even when institutional services meet formal criteria, they may be judged as moral failure if they contradict shared expectations of filial responsibility [35, 41]. As a result, institutional legitimacy must be negotiated not only individually but collectively.

Together, these mechanisms of role alignment, emotional resonance, and public endorsement form the core channels through which family ethics are translated into institutional legitimacy. Since this translation involves complex cultural and psychological shifts that extend beyond simple linear causality, this study explores the process through the following qualitative proposition.

*Proposition 1 (P1): The quality of the institutional translation of family ethics significantly determines the level of initial trust and moral legitimacy rural older adults accord to digital care institutions.*

### Digital ethics as the cultural tension field of institutional transformation

Even when institutional care achieves moral recognition, behavioral adoption remains uncertain. In rural settings, many older adults who affirm the legitimacy of digital services still resist their use [18, 23]. This disjunction reflects a deeper tension between institutional structure and cultural interpretation, particularly regarding how care is operationalized in digital systems.

Digital elder care increasingly relies on algorithmic decision-making, standardized protocols, and efficiency-based metrics [9]. While these logics improve technical coordination, they often marginalize the relational and symbolic dimensions of care. Mol [26] and Verloo [38] note that such rationalized systems may suppress the emotional experiences central to older adults’ moral judgments.

To bridge this gap, this study adopts an extended Technology Acceptance Model (TAM), focusing on perceived usefulness (PU) and perceived ease of use (PEU) as mediating variables [8, 37]. PU reflects older adults’ assessment of whether digital care enhances their well-being, while PEU captures their confidence in navigating digital interfaces. Recent extensions to TAM emphasize the role of emotion, social trust, and cultural congruence in influencing digital engagement [29, 40]. These findings suggest that institutional legitimacy must be perceptually translated into personally meaningful and operationally feasible experiences.

In the conceptual model of this study, the ethical evaluations of privacy, dignity, and social consensus do not only directly shape intentions but also operate through the cognitive mediation of technology acceptance. When older adults perceive that digital services respect their moral boundaries and emotional needs, they are more likely to find the technology easier to master and more beneficial to their daily lives. Therefore, the study proposes the following mediation hypotheses.

*H2a: Perceived ease of use mediates the relationship between privacy and data protection and behavioral intention.*

*H2b: Perceived ease of use mediates the relationship between dignity in care and behavioral intention.*

*H2c: Perceived ease of use mediates the relationship between care acceptability and behavioral intention.*

*H3: Perceived ease of use positively influences perceived usefulness, which in turn positively affects behavioral intention.*

### Ethical lag as an explanatory path for stratified technical behavior

Technological transformation does not unfold uniformly across social strata. Ogburn’s [27] theory of cultural lag explains how rapid advancements in material culture often outpace adaptations in non-material culture, including ethical norms and value systems. This gap generates a structural lag wherein individuals’ moral frameworks remain out of sync with institutional innovations. In rural elder care, this manifests as an “ethical lag”: digital platforms evolve swiftly, but the normative structures guiding older adults’ moral reasoning, grounded in kinship, ritual, and relational trust, adjust more slowly.

In this context, technology adoption is not merely a question of rational choice but a negotiated process of ethical and emotional realignment. The Stimulus–Organism–Response (S–O–R) model [25] offers a more nuanced framework: behavior is shaped not by stimulus alone, but by internal processing of emotional resonance, moral congruence, and social evaluation [3, 17]. Ethical lag thus operates not passively but as an active tension within this translation chain.

**Fig. 1:**
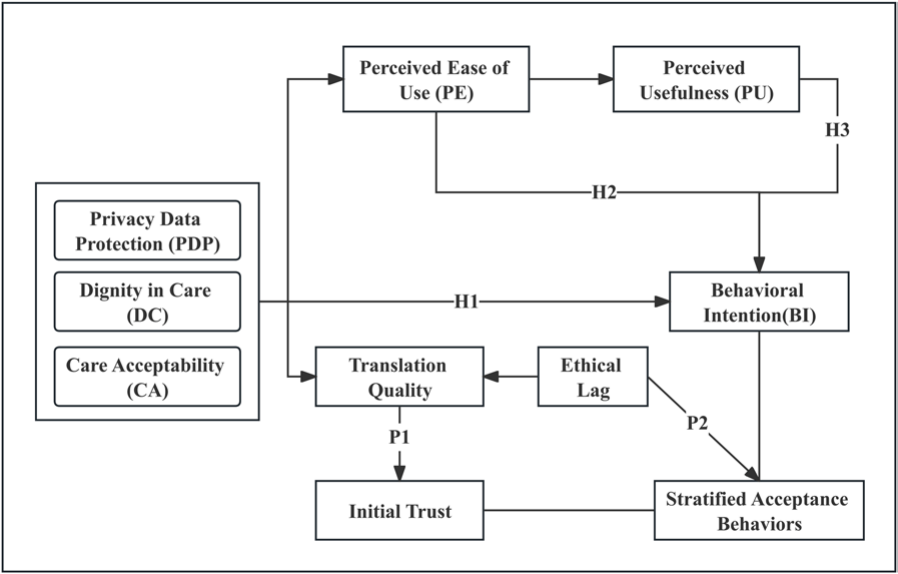
Framework

Crucially, ethical lag leads to stratified adoption patterns. Some older adults, equipped with higher digital literacy, stronger family support, or greater institutional trust, adapt more readily. Others disengage, not due to hostility but due to unresolved ethical dissonance. This produces differentiated response paths, accelerating existing social inequalities within service provision. Ethical lag, then, becomes not merely a temporal delay but an institutional mechanism that stratifies digital transformation outcomes. Based on these qualitative insights, this study proposes:

*Proposition 2 (P2): Ethical lag, specifically the gap between rapid technological intervention and the slower adaptation of traditional filial norms, leads to stratified acceptance behaviors and differentiated adoption paths among rural older adults *(Fig 1)*.*

## Research design and methods

This study adopts a mixed methods design that integrates quantitative and qualitative approaches to provide a comprehensive understanding of technology adoption in rural elder care. The quantitative strand is designed to test the hypothesized causal relationships between specific ethical dimensions, technological perceptions, and behavioral intentions through structural equation modeling.

In contrast, the qualitative strand aims to explore the nuanced cognitive affective mechanisms and moral dilemmas that are often inaccessible through standardized survey instruments. By utilizing semi structured interviews, the study delves into the processes of institutional translation and the tensions of ethical lag that shape the decision making of older adults. This dual approach ensures that the research captures both the breadth of behavioral trends and the depth of cultural experiences in the digital transformation of rural social welfare.

### Sampling and participants

The empirical site for this study is Shapingba District in Chongqing, China, a municipality with one of the highest aging rates in the country (17.08% aged 65 and above according to the 2021 Census) and significant intra-district variation in economic development. This location provides a representative context for studying rural digital elder care under differentiated institutional and infrastructural conditions. Using a stratified sampling strategy, five township elder care service stations were selected to represent three types of regions: urban–rural fringe areas (e.g., Fenghuang Town, Huilongba Town), economically active areas (e.g., Geleshan Town, Zhongliang Town), and remote underdeveloped areas (e.g., Qingmuguan Town). From each township, 90 older adults aged 60 and above with sufficient cognitive ability were recruited to ensure their capacity to articulate perceptions of digital care services. This yielded 450 initial responses, of which 400 valid samples were retained after screening, resulting in an effective rate of 93.89%. A priori power analysis conducted using G*Power 3.1 (with f^2^ = 0.15, α = 0.05, power = 0.805) confirmed the statistical adequacy of the sample. Additionally, 15 participants were purposively selected from the survey pool for follow-up semi-structured interviews, based on variation in institutional attitudes, prior technology exposure, and caregiving arrangements.

### Data collection and processing

Data were collected through a dual-channel approach. The quantitative survey was administered both online (via Credamo with voice narration) and offline, with support from trained facilitators to assist elderly participants. The questionnaire incorporated logical skip patterns and consistency checks to enhance data quality. For the qualitative component, in-depth interviews were conducted face-to-face using a semi-structured protocol that explored caregiving expectations, emotional experiences with smart services, sources of trust or hesitation, and narratives of resistance or dependence (See Table 1).

**Table 1:** Semi-structural interview outline

Theme	Content
Service Awareness and Initial Assessment	Are you aware that community-based staff or personnel in nursing centers provide services for the elderly, such as meal delivery, blood pressure monitoring, and assistance with bathing and daily care?
	Have you personally utilized or experienced such services? If yes, could you describe in detail the specific services you have received?
	Have you heard of township-level elderly care service centers? If so, what is your understanding of the types of support or services they provide?
	Are you aware that some elderly individuals have access to home-based devices, such as emergency call systems or health monitoring equipment? In your opinion, how useful or beneficial do you consider these devices to be?
Moral Framing of Care: Family vs. Community	In the past, elder care was mainly provided by family members. Now, community organizations and elderly care service centers are also becoming involved. How do you perceive or feel about this shift in the source of care?
	If your family encourages you to use smart devices to assist in daily life, would you regard this primarily as a matter of convenience, or would you interpret it as a reduction in family care and attention?
Role of Informal Support Networks	Do you have neighbors who regularly support you, for example by helping with small tasks or engaging in conversation? How important is this kind of mutual assistance in your daily life?
	Would you feel comfortable if community services or smart devices were to replace some of this neighborly support? Why or why not?
Perceived Relational Quality of Digital Care	To what extent do you believe that technological devices (such as health monitoring systems or emergency call devices) can substitute for human care? Please explain your view
	If such devices make you feel emotionally distant or less cared for, would you still be willing to use them? Why or why not?

All interviews were audio-recorded, transcribed verbatim, and coded using MAXQDA 2024.3. The qualitative analysis followed the Gioia methodology to ensure systematic inductive rigor [14]. This involved coding raw data into 1 st order concepts, which were then synthesized into 2nd order themes and ultimately grouped into aggregate dimensions to construct a grounded data structure (Table 2).

**Table 2:** Profile of interview participants

No	Gender	Age	Lifestyle	No	Gender	Age	Lifestyle
1	Female	72	Home-based care	9	Female	75	Institutional care
2	Female	66	Home-based care	10	Female	83	Institutional care
3	Female	63	Home-based care	11	Male	73	Home-based care
4	Female	84	Institutional care	12	Female	82	Institutional care
5	Male	90	Institutional care	13	Female	78	Institutional care
6	Male	88	Institutional care	14	Male	81	Institutional care
7	Male	90	Institutional care	15	Female	76	Institutional care
8	Female	82	Institutional care				

### Variable operationalization and model construction

The measurement instrument originated from a comprehensive baseline survey on rural digital transformation. To ensure content validity regarding ethical and cognitive drivers, a pilot study (*N* = 30) was conducted in December 2024 to bridge linguistic and conceptual gaps. Results indicated that abstract academic terminology conflicted with the cognitive habits of rural seniors, prompting a refinement of the 79-item survey into 55 items by merging redundant paths and rephrasing questions into culturally intelligible language.

Specific optimizations involved replacing formal prompts with grounded scenarios. For instance, an abstract item regarding personal dignity was replaced with a relatable question on whether emotional neglect is felt when digital monitoring replaces in person care. Furthermore, repetitive items concerning equipment costs were consolidated into the Care Acceptability construct to reduce respondent fatigue. Within this refined instrument, the current model was tested using 15 targeted items covering three independent ethical constructs, technological perceptions, and behavioral intentions (see Table 3).

**Table 3:** Questionnaire and variable setting

Construct	Item Description
Privacy and Data Protection	Q4.2. Would you consider it an invasion of privacy if the community provided free health-monitoring bracelets? Q4.4. How important is the protection of your health data privacy to you?
Dignity in Care	Q4.3. Do you feel disrespected when communicating with a doctor through video consultation? Q4.19. Do you feel lonely or emotionally neglected if care is delivered mainly through digital equipment rather than in person?
Care Acceptability	Q4.6. Do you agree that strict protection of health data privacy should be a shared consensus among the elderly in the community? Q4.17. Do you believe that managing health through digital devices represents the future of elderly care and should be accepted as much as possible?
Perceived Ease of Use	Q1.19. Do you currently use electronic devices such as smartphones? Q5.5. Do you feel nervous or uncomfortable when using smart devices?
Perceived Usefulness	Q3.3. Would you be willing to use a free smart device to monitor your health? Q3.21. If nursing homes combined activities with digital elderly care (e.g., guidance on smart device use, health management courses, online fitness tutorials), would you be willing to participate? Q3.5. If community service centers or other organizations provided free health lectures or recreational activities (such as aerobics, nutrition classes) via mobile phones or TV, would you participate? Q3.10. Would you be willing to participate in online health lectures or virtual recreational activities?
Behavioral Intention	Q3.15. Would you be willing to rely primarily on digital devices to manage your retirement life? Q3.22. Do you think that providing digital elderly care services (e.g., smart wristbands, health monitoring, social media apps, or remote health consultations) could motivate you to engage more actively in community or nursing home activities?

Privacy and Data Protection, Dignity in Care, and Care Acceptability are modeled as three distinct first-order constructs. The items for Perceived Ease of Use, Perceived Usefulness, and Behavioral Intention were adapted from Davis [8] and contextualized for digital eldercare and rural older adults

Three independent ethical constructs served as primary predictors: Privacy and Data Protection (PDP), reflecting the sanctity of the domestic sphere; Dignity in Care (DC), capturing emotional quality in tech mediated care; and Care Acceptability (CA), assessing communal endorsement. Items were adapted with reference to Rest (1986) and further contextualized for rural digital eldercare settings. Perceived Usefulness (PU) was measured by four items adapted from Davis [8], while Perceived Ease of Use (PE) and Behavioral Intention (BI) each comprised two items. The structural equation model (SEM) is specified as:

$$\begin{array}{c}\mathrm{P}\mathrm{U}={\mathrm{a}}_{1}\mathrm{P}\mathrm{E}+{\upvarepsilon}_{1}\\ \mathrm{P}\mathrm{E}={\upbeta}_{1}\mathrm{P}\mathrm{D}\mathrm{P}+{\upbeta}_{2}\mathrm{D}\mathrm{C}+{\upbeta}_{3}\mathrm{C}\mathrm{A}+{\upvarepsilon}_{2}\\ \mathrm{B}\mathrm{I}={\upgamma}_{1}\mathrm{P}\mathrm{U}+{\upgamma}_{2}\mathrm{P}\mathrm{E}+{\upgamma}_{3}\mathrm{P}\mathrm{D}\mathrm{P}+{\upgamma}_{4}\mathrm{D}\mathrm{C}+{\upgamma}_{5}\mathrm{C}\mathrm{A}+{\upvarepsilon}_{3}\end{array}$$

where α, β, and γ represent path coefficients and ε denotes error terms.

## Empirical analysis

### Descriptive analysis of sample and coded data

#### Data purification and imputation

To ensure sample integrity, a multi-stage protocol was applied to 450 initial responses. First, 23 duplicates were removed, including staff test surveys (*n* = 13) and double submissions (*n* = 10). An additional 27 responses were excluded due to response times under 2 min (*n* = 11), identical IP addresses (*n* = 9), or unverifiable logical contradictions (*n* = 7). This resulted in a final analytical sample of 400 valid responses. Within these cases, 32 records containing sporadic missing values were addressed via Multiple Imputation by Chained Equations (MICE) under a Missing at Random (MAR) assumption. SEM parameters were estimated by pooling results from five complete datasets using Rubin’s rules to propagate imputation uncertainty. To prevent artificial variance shrinkage, logical inference was strictly restricted to demographic variables and never applied to latent scale items.

#### Quantitative sample profile and measurement model validation

The demographic profile (*N* = 400) represents a deeply aging rural population: 42.5% male, 57.5% female, and 39.5% aged 80 or above. Educational attainment is notably low (94.9% below high school), and 43% reside in institutional settings (Table 4).

**Table 4:** Demographic profile of respondents (*N* = 400)

Characteristic	Category	Frequency (N)	Percentage (%)
Gender	Male	170	42.5
	Female	230	57.5
Age	60–69	111	27.7
	70–79	131	32.8
	≥ 80	158	39.5
Income	National insurance	177	44.3
	Child support	59	14.8
	Post-retirement work	59	14.7
	Financial management, etc	43	10.7
	Personal savings	43	10.7
	Other	19	4.8
Lifestyle	Home-based care	226	56.5
	Institutional care	172	43
	Other	2	0.5
Health	Very healthy	101	25.2
	Occasional discomfort	114	28.5
	Fair health, needs help	69	17.3
	Not very healthy	64	16
	Requires long-term care	52	13
Proficiency	Cannot use at all	111	27.7
	Basic functions only	57	14.2
	Can use common functions	116	29
	Can use most functions	71	17.8
	Proficient in using	45	11.3

Income = Income Source; Health = Health Status; Proficiency = Proficiency with Electronics

Income = Income Source; Health = Health Status; Proficiency = Proficiency with Electronics

The reliability and validity of the measurement model were confirmed (see Tables 5 and 6). All standardized factor loadings were significant and generally acceptable. Cronbach’s alpha values ranged from 0.710 to 0.903, indicating overall acceptable internal consistency. Most CR and AVE values met or closely approached the recommended thresholds of 0.70 and 0.50, respectively. Although the CR for Privacy and Data Protection (PDP) was slightly below 0.70 (CR = 0.687), it remained acceptable given the exploratory nature of the construct and the contextualized elderly sample. Furthermore, discriminant validity was established as the square root of the AVE for each construct exceeds its correlations with other constructs. Correlation analysis (Table 7) confirms theoretical expectations, with the strongest association between Perceived Ease of Use and Behavioral Intention (r = 0.654, *p* < 0.001). Robustness checks (see Appendix Table A3) confirm that their retention did not significantly bias these path coefficients, allowing them to be utilized as key informants for the subsequent qualitative strand.

**Table 5:** Results of the measurement model

Construct	Item	Standardized Loading	Alpha	CR	AVE
Privacy Data Protection (PDP)	PDP1	0.709	0.710	0.687	0.524
	PDP2	0.739			
Dignity in Care (DC)	DC1	0.896	0.835	0.827	0.706
	DC2	0.780			
Care Acceptability (CA)	CA1	0.779	0.758	0.754	0.605
	CA2	0.777			
Perceived Ease of Use (PE)	PE1	0.791	0.781	0.761	0.615
	PE2	0.777			
Perceived Usefulness (PU)	PU1	0.852	0.903	0.909	0.714
	PU2	0.835			
	PU3	0.849			
	PU4	0.845			
Behavioral Intention (BI)	BI1	0.779	0.762	0.738	0.585
	BI2	0.750			

*N* = 400. All standardized factor loadings were significant at the *p* < 0.001 level *CR* Composite reliability, *AVE* Average variance extracted. Fit indices: χ^2^/df = 1.105, CFI = 0.990, TLI = 0.987, RMSEA = 0.016, SRMR = 0.028

**Table 6:** Discriminant validity using the Fornell–Larcker criterion

Construct	PDP	DC	CA	PE	PU	BI
PDP	**0.724**					
DC	0.584	**0.840**				
CA	0.542	0.692	**0.778**			
PE	0.456	0.357	0.506	**0.784**		
PU	0.308	0.241	0.342	0.677	**0.845**	
BI	0.535	0.473	0.639	0.664	0.640	**0.765**

*N* = 400. Diagonal elements (bold) represent the square root of the Average Variance Extracted (AVE) for each construct. Off-diagonal elements represent the Pearson correlation coefficients between constructs. Discriminant validity is established when the diagonal elements are greater than the off-diagonal elements in the corresponding rows and columns

**Table 7:** Descriptive statistics and correlations

Variable	Mean	SD	PDP	DC	CA	PE	PU	BI
Privacy and Data Protection (PDP)	3.125	0.639	1					
Dignity in Care (DC)	3.061	0.788	0.448***	1				
Care Acceptability (CA)	3.139	0.746	0.390***	0.543***	1			
Perceived Ease of Use (PE)	2.714	0.724	0.350***	0.299***	0.414***	1		
Perceived Usefulness (PU)	3.219	0.755	0.180***	0.156**	0.193***	0.583***	1	
Behavioral Intention (BI)	3.120	0.681	0.367***	0.369***	0.460***	0.654***	0.572***	1

*N* = 400. *** *p* < 0.001

#### Qualitative coding and saturation

To maximize the explanatory power of the mixed-methods design, the 15 outliers identified in the quantitative phase were intentionally selected as the primary source for the qualitative strand. This extreme case sampling allowed for a deep exploration of unique ethical dissonances that deviate from general statistical patterns. Following the Gioia methodology, the three-stage inductive process distilled raw interview data from these cases into 891 1st-order concepts, 16 2nd-order themes, and 5 aggregate dimensions: Moral Conviction (AD1), Initial Trust (AD2), Perceptual Processing (AD3), Behavioral Response (AD4), and Tension Nodes (AD5).

**Fig. 2:**
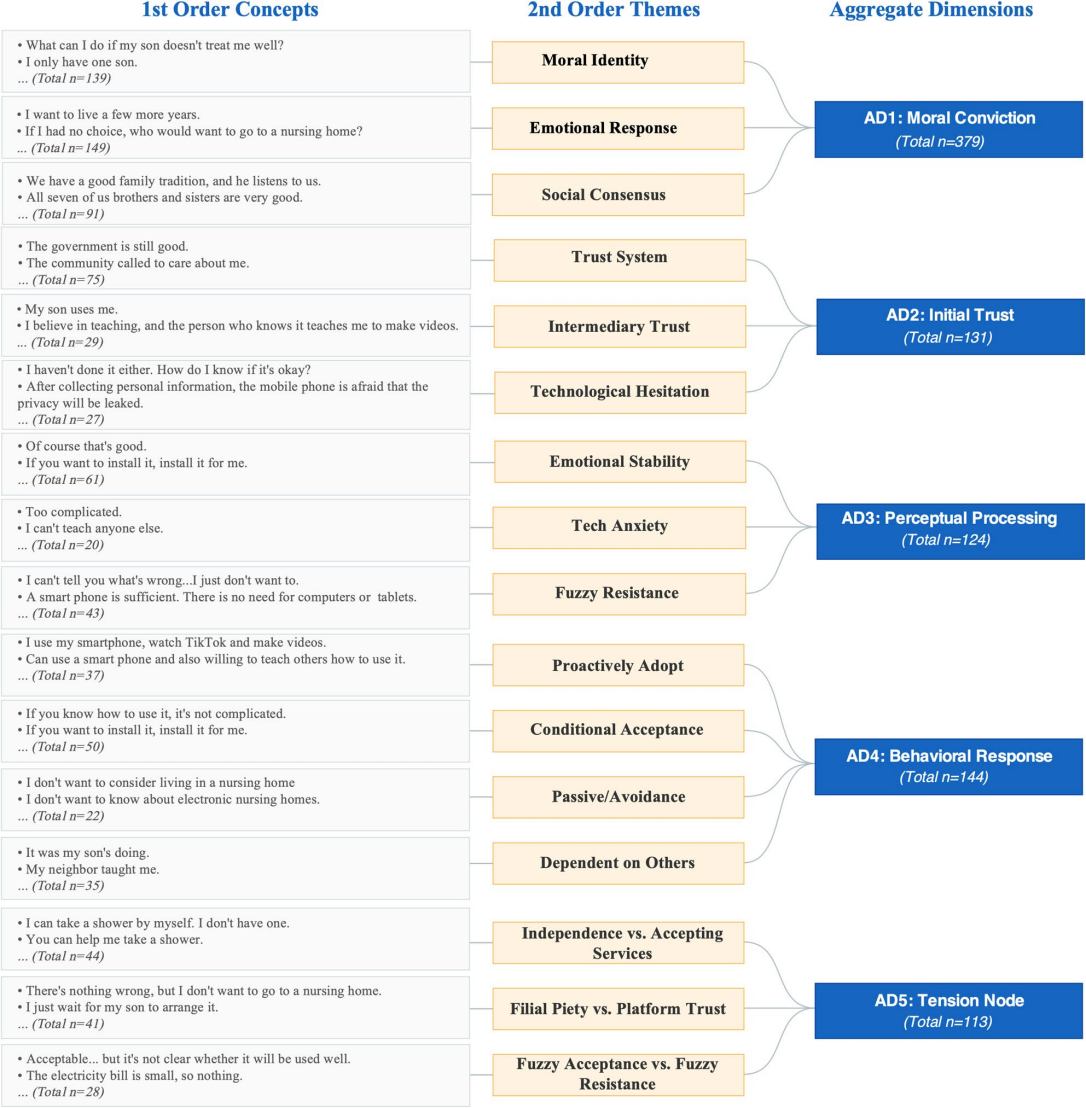
Gioia data structure of qualitative coding

Theoretical saturation was reached when no new 1st-order concepts emerged from the final three interviews. To ensure interpretive reliability, two researchers conducted independent coding. Discrepancies were resolved through collaborative reconciliation and consultation with a third senior researcher until 100% consensus was reached (Fig. 2)

### Quantitative analysis of ethical dimensions and behavioral intention

Structural equation modeling (SEM) was employed to test how Privacy Data Protection (PDP), Dignity in Care (DC), and Care Acceptability (CA) influence usage intentions through the technology acceptance framework. This analysis clarifies how specific ethical dimensions associate with digital eldercare adoption intention. The full correlation matrix, including demographic controls, is detailed in Appendix Table A1 to ensure foundational data transparency.

**Fig. 3:**
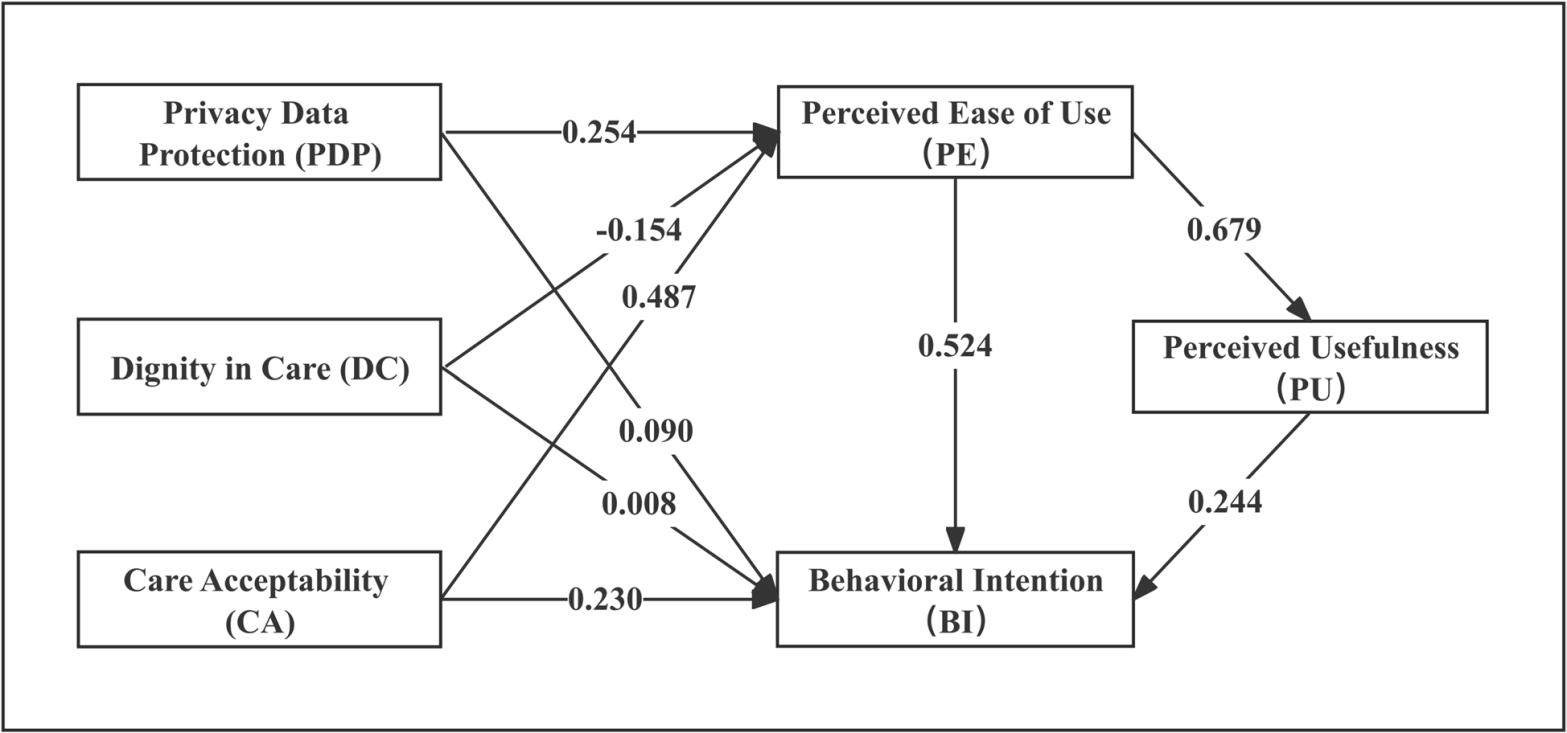
SEM of the behavioral intention

As indicated in Table 8, ethical dimensions exert varying degrees of influence on Behavioral Intention (BI). Care Acceptability stands out as a critical driver, exerting a marginally significant direct positive effect on BI (β = 0.230, *p* < 0.1), suggesting that social and ethical endorsement within rural communities directly bolsters individual engagement. Conversely, direct paths from Privacy Data Protection and Dignity in Care to BI were not statistically significant (*p* > 0.1). However, total effects in Table 9 reveal a more nuanced dynamic; while PDP lacks a direct link, its total effect on BI is significant at 0.265, confirming its impact is fully realized through indirect cognitive appraisals. In contrast, Dignity in Care showed no significant direct or total effect (*p* = 0.341), implying that individualized dignity concerns may be overshadowed by functional necessity and collective social norms in rural China. These relationships remained consistent when controlling for age, gender, and digital proficiency (Appendix Table A2) (Fig. 3)

**Table 8:** Results of path analysis

Path	Estimate (β)	S.E	z-value	*p*-value
PDP → PE	0.254**	0.114	2.811	0.005
DC → PE	−0.154	0.129	−1.509	0.131
CA → PE	0.487***	0.135	4.567	0.000
PE → PU	0.679***	0.087	8.726	0.000
PDP → BI	0.090	0.181	1.293	0.196
DC → BI	0.008	0.197	0.112	0.911
CA → BI	0.230*	0.247	2.430	0.015
PE → BI	0.524***	0.292	3.705	0.000
PU → BI	0.244**	0.144	3.147	0.002

*β* Standardized path coefficient, *S.E.* Standard Error. Variable *Abbreviations*: *PDP* Privacy Data Protection, *DC* Dignity in Care, *CA* Care Acceptability, *PE* Perceived Ease of Use, *PU* Perceived Usefulness This table tests the significance of individual paths

^*^p < 0.10, **p < 0.01, *** *p* < 0.001

**Table 9:** Results of direct, indirect, and total effects

Path	Standardized Effect	S.E	z-value	*p*-value	95% Boot CI
Total Effect					
PDP → BI	0.265**	0.091	2.908	0.004	[0.074, 0.435]
DC → BI	−0.098	0.103	−0.951	0.341	[−0.305, 0.095]
CA → BI	0.566***	0.097	5.814	< 0.001	[0.379, 0.761]
Total Indirect					
PDP → PE → BI	0.133*	0.057	2.329	0.020	[0.027, 0.256]
DC → PE → BI	−0.081	0.060	−1.348	0.178	[−0.209, 0.029]
CA → PE → BI	0.255***	0.073	3.492	< 0.001	[0.135, 0.425]
Indirect Effect					
PE → PU → BI	0.166**	0.051	3.234	0.001	[0.056, 0.262]

Note: *PDP* Privacy Data Protection, *DC* Dignity in Care, *CA* Care Acceptability, *PE* Perceived Ease of Use, *PU* Perceived Usefulness, *BI* Behavioral Intention. *S.E.* Standard Error, *CI* Confidence Interval. 95% Confidence Interval based on 5,000 bootstrap resamples. An effect is considered significant if the 95% confidence interval does not contain zero

^*^p < 0.10, **p < 0.01, *** *p* < 0.001

The analysis further elucidates the translation of ethical dimensions into BI via Perceived Ease of Use (PE) and Perceived Usefulness (PU). Both PDP (β = 0.254, *p* = 0.005) and CA (β = 0.487, *p* < 0.001) significantly enhance PE, suggesting that ethical alignment reduces cognitive friction and emotional alienation. Notably, a robust sequential mediation chain emerged from Perceived Ease of Use through Perceived Usefulness to BI. Perceived Ease of Use exerts a powerful influence on Perceived Usefulness (β = 0.679, *p* < 0.001), which subsequently drives BI at 0.244. This cascading dynamic underscores that ensuring ethical acceptability is a vital prerequisite for recognizing practical utility.

To confirm statistical robustness, nonparametric bootstrap procedures with 5,000 resamples generated bias-corrected confidence intervals (CIs). As reported in Table 9, indirect effects from privacy data protection through perceived ease of use to behavioral intention, and from care acceptability through perceived ease of use to behavioral intention, are both significant. The sequential mediation from perceived ease of use through perceived usefulness to behavioral intention was also supported. Furthermore, stability testing against alternative structural configurations (Appendix Table A3) confirms that path coefficients and fit indices remain highly stable.

### Qualitative typology of ethical translation and behavioral responsiveness

This section explores ethical translation mechanisms by synthesizing coding results from fifteen outlier cases. Following a developmental logic from specific concepts to integrated patterns, the analysis first unpacks dimensional associations of ethical lag before culminating in a formal typology.

**Fig. 4:**
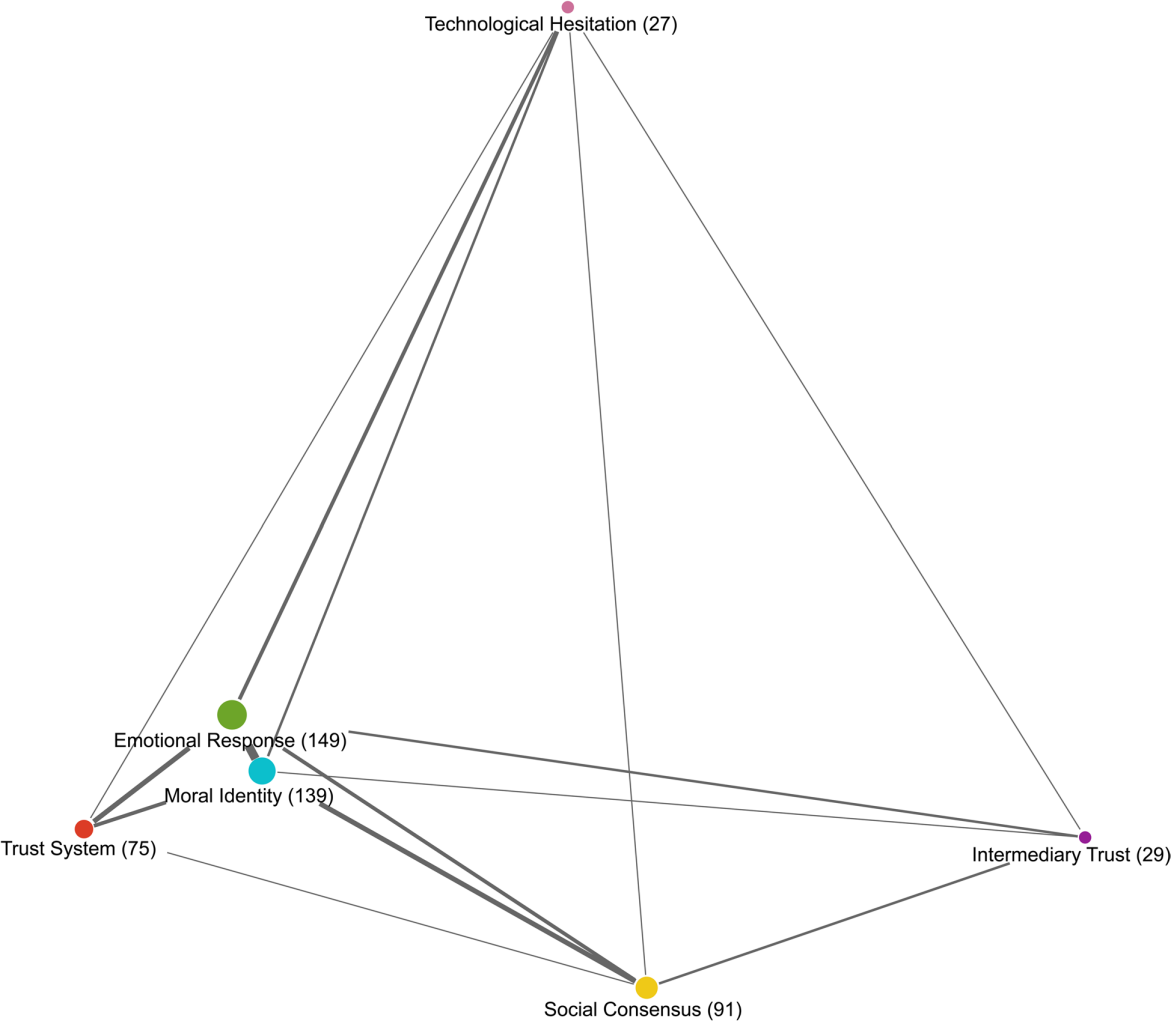
Co-occurrence network based on interview segments. Based on coded interview segments, nodes represent themes (frequencies in parentheses), grouped into moral conviction (e.g., Moral Identity) and initial trust (e.g., Trust System). Edge thickness reflects co-occurrence frequency. No minimum threshold was imposed to preserve the structural interdependencies of all core themes

The analysis utilized a Gioia methodology to distill narratives into five aggregate dimensions: moral conviction, initial trust, perceptual processing, behavioral response, and tension nodes. To explore relational dynamics, a co-occurrence network was conducted (Fig. 4), mapping the conceptual proximity between moral identity, trust systems, and technological hesitation. High-density associations between moral identity and emotional response suggest technology is evaluated through an affective lens of filial piety. Specifically, when technology threatens familial duty, moral identity co-occurs intensely with tension nodes, while role-delegation concepts cluster with adaptive processing. To ensure comprehensive representation, no minimum co-occurrence threshold was imposed, preserving the full structural interdependencies.

**Fig. 5:**
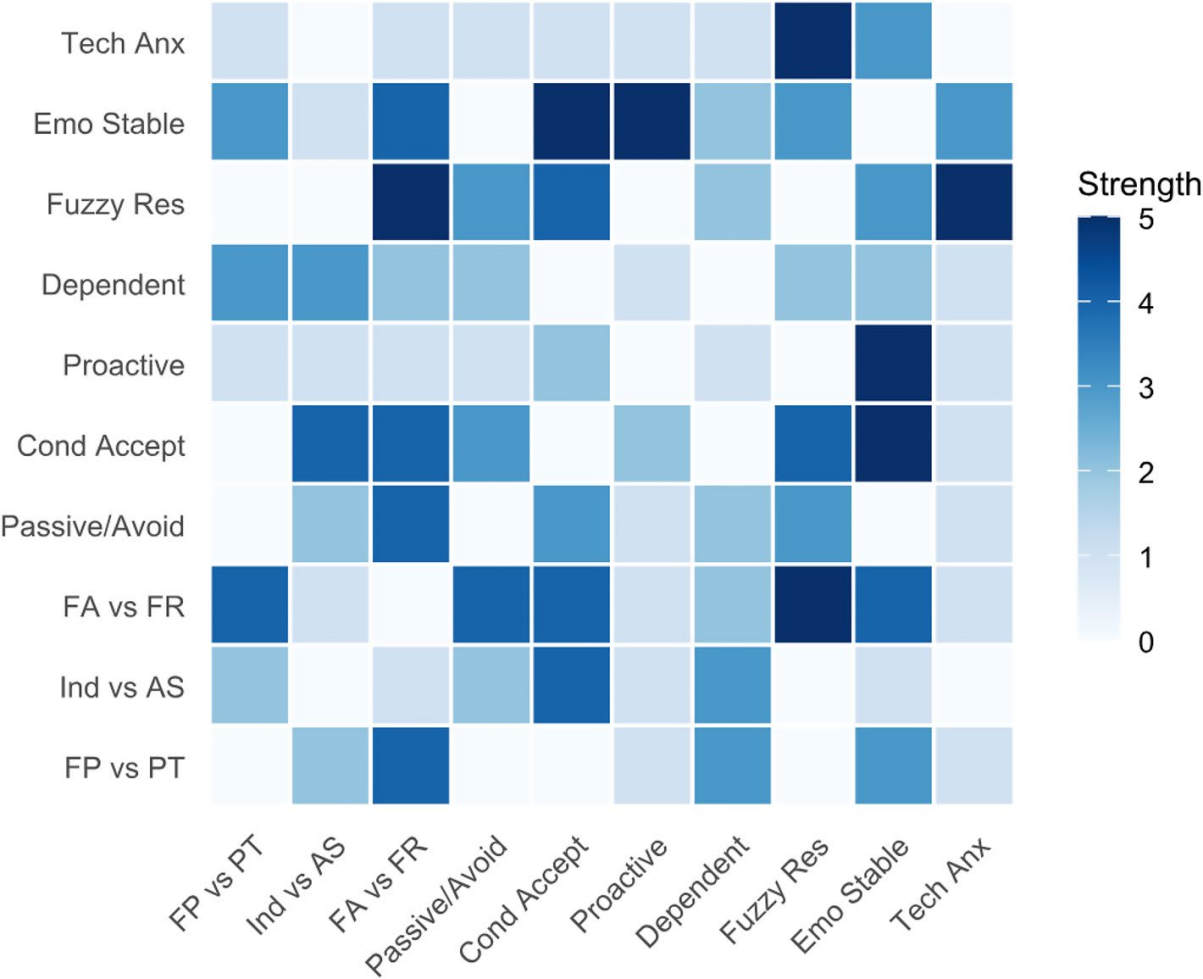
Typological mapping. This heatmap visualizes association strengths between tension nodes, behavioral responses, and perceptual processing on a 0–5 scale (darker cells indicate stronger links). Derived from the code matrix of interview segments, no minimum threshold was applied to present the full relational landscape

Building on these associations, a typological mapping (Fig. 5) visualized relationship intensities. This heatmap, using a 0–5 association scale, delineates psychological adjustment zones between normative discomfort and reactive dependency, bridging raw codes and theoretical categories. Based on this, a formal typology was synthesized using specific decision rules (Table 10).

**Table 10:** Qualitative typology and decision rules

Typology Pattern	Decision Rules for Assignment	Illustrative Behavioral Logic	Representative Evidence
Category A: Moral-Trust Congruence (smooth translation)	*Criteria:* Positive AD1 (Moral Conviction) + High AD2 (Initial Trust) + High AD3 (Perceptual Processing) + Positive AD4 (Behavioral Response) + Low AD5 (Tension Node)	*Smooth Translation:* These participants experience high alignment between digital care and family ethics. They utilize intergenerational support to bridge the digital divide, viewing platforms as tools that enhance filial piety	*"My son and my grandson want to teach me… It’s better than being at home." (ID: 9–241)*
			*"Living in a nursing home is inevitable… and acceptable." (ID: 5–23)*
Category B: Moral-Trust Conflict (translation failure)	*Criteria:* Conflictive AD1 (Moral Conviction) + Low AD2 (Initial Trust) + Negative AD3 (Perceptual Processing) + Avoidant AD4 (Behavioral Response) + High AD5 (Tension Node)	*Translation Failure:* A significant Ethical Lag exists here. Strong traditional moral identity creates a massive tension node with platform care. Resistance is a choice to preserve moral identity against perceived institutional intrusion	*"It’s in our family’s genes… It just doesn’t feel right [to use platforms]." (ID: 1–147)*
			*"No one wants to go, but sometimes there’s no one else to care for you." (ID: 1–255)*
Category C: Moral-Trust Ambiguity (transition period)	*Criteria:* Ambivalent AD1 (Moral Conviction) + Conditional AD2 (Initial Trust) + Ambivalent AD3 (Perceptual Processing) + Dependent AD4 (Behavioral Response) + High AD5 (Tension Node)	*Ambiguous Acceptance:* These participants exist in a "transition period". Due to the empty-nesting reality in rural areas, they are forced to use services out of necessity (AD4: Dependency) while emotionally retaining moral discomfort	*"There’s no one left at home… I’ve gotten used to it [out of necessity]." (ID: 6–69)*
			*"I’ll call them when I can’t manage anymore." (ID: 3–47)*

Category A: Moral-Trust Congruence (Smooth Translation) represents participants with high alignment between digital care and family ethics. The logic is one of smooth translation, where older adults use intergenerational support to bridge the digital divide, viewing platforms as tools enhancing filial piety. Case 9–241 exemplifies that family-supported learning makes institutional care more acceptable than isolation (9–241, 5–23). This category is assigned when positive moral convictions (AD1) and high trust (AD2) coincide with positive behavioral responses (AD4) and minimal tension (AD5).

Category B: Moral-Trust Conflict (Translation Failure) reveals significant ethical lag where traditional identity creates massive tension with institutional care. The logic is characterized by translation failure, where resistance preserves moral identity against perceived intrusion (1–147, 1–255). This pattern is identified when conflictive convictions (AD1) and low trust (AD2) trigger negative processing (AD3) and high tension (AD5).

Category C: Moral-Trust Ambiguity (Transition Period) reflects a transition period driven by rural empty-nesting. The logic is defined as ambiguous acceptance, where participants use services out of necessity while retaining moral discomfort (6–69, 3–47). Decision rules define this by ambivalent positions (AD1) and conditional trust (AD2) paired with dependent responses (AD4) and persistent tension (AD5).

The synthesis of these categories provides empirical evidence for the research propositions. For Proposition 1, the Category A-B contrast demonstrates that institutional trust is contingent upon ethical translation efficacy. Trust is affirmed through moral resonance but obstructed by normative dissonance during translation failure. For Proposition 2, Category C confirms that moral lag, arising from mismatched individual and institutional frameworks, drives stratified behavioral responsiveness. Rather than a binary state, behavioral intention is a negotiated outcome of this ethical lag. Thus, ethical resonance determines whether digital care is interpreted as an empowerment of filial piety or an intrusion upon traditional identity.

### Methodological integration and robustness

This study utilized an integrative validation approach bridging statistical patterns with deep-case analysis.Quantitative robustness was established by confirming the dataset's structural suitability for factor analysis, yielding a Kaiser–Meyer–Olkin (KMO) measure of 0.891 and a significant Bartlett’s test (*p* < 0.001). Beyond the high internal consistency reported in Table 5, stability testing against alternative structural configurations (Appendix Table A3) confirmed that path coefficients remained highly consistent and were not biased by the retention of outlier samples.

Methodological convergence was achieved by purposively selecting 15 outliers from the quantitative phase for follow-up interviews to address dissonances unexplained by the general model. In this strand, coding stability was reached from the 13th interview onward, as no new 1st-order concepts emerged across the 16 secondary themes.

### Ethical lag and stratified responsiveness: a contextual explanation

This section theorizes ethical lag as a perceived misalignment occurring when the rapid expansion of digital welfare outpaces the adaptation of localized care expectations. In rural China, this lag is empirically manifested through specific psychosocial concerns regarding the sanctity of private domestic spaces and the maintenance of personal dignity during tech-mediated interactions. Consequently, resistance to digital eldercare is interpreted through value systems where institutional interventions are assessed based on their compatibility with family-centered care boundaries. Adoption decisions are thus characterized as pragmatic assessments of moral legitimacy, conditioned by factors such as prior institutional trust and the potential displacement of traditional family roles.

Across this terrain, three distinct patterns of responsiveness emerge from the data. Adaptive translation involves the selective reframing of care expectations to accommodate institutional support, often facilitated by intergenerational mediation. Ethical dissonance arises from unresolved tensions between individual expectations of care dignity and the standardized, efficiency-based design of digital services. Finally, translation failure occurs when institutional cues lack cultural legibility, prompting older adults to disengage as a means of preserving their normative identity. These patterns suggest that ethical lag functions as a stratifying mechanism, distinguishing individuals capable of aligning personal care frameworks with digital logic from those who remain excluded despite formal service access.

**Fig. 6:**
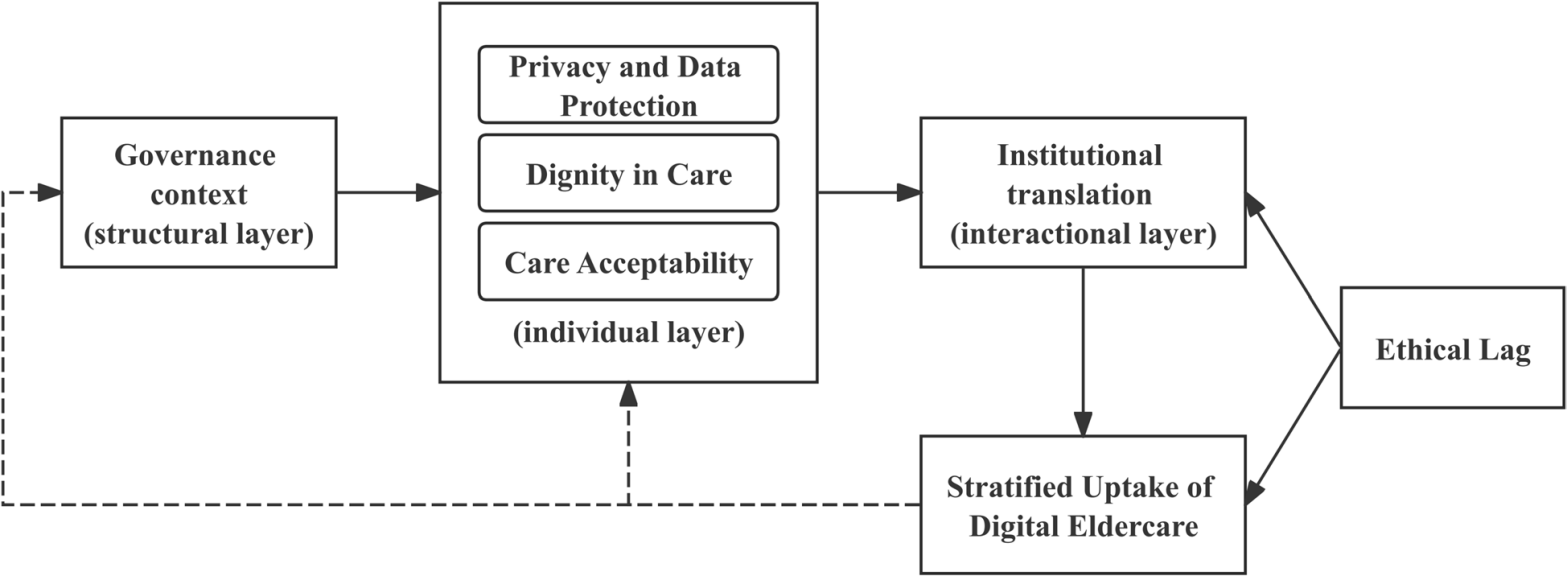
Stratified responsiveness framework

To provide a systematic tool for service evaluation, this study proposes the Stratified Responsiveness Framework (SRF) illustrated in Fig. 6. At the individual layer, core perceptions of privacy and dignity shape how technology is deemed legitimate. The interactional layer focuses on institutional translation, reflecting the degree to which service design respects or neglects these specific ethical expectations. Finally, the structural layer encompasses the broader governance context, including trust networks and spatial inequalities that enable or constrain ethical alignment. This framework suggests that in rapidly transforming welfare states, policy success hinges not only on technical accessibility but on ethical fit, representing the resonance between digital instruments and the lived moral ecologies of service users.

## Conclusion

### Conclusion

This study investigates the psychosocial and ethical factors shaping the willingness of rural older adults in China to adopt digital public eldercare services. Utilizing a mixed-methods design, the research demonstrates that technology adoption is not merely a rational or technical choice but is mediated by perceptions of ethical legitimacy. When digital care services are perceived as aligned with role expectations, emotional needs for dignity, and communal values of care, they become a viable alternative to traditional family-based arrangements. Conversely, perceived misalignment regarding privacy boundaries and the nature of mediated interactions generates hesitation, resistance, or complete disengagement, regardless of technical accessibility or affordability.

Rather than attributing such resistance to digital illiteracy or cultural inertia, this study introduces the concept of ethical lag to explain the discordance between rapid digital welfare reforms and embedded care expectations. Ethical lag functions as a stratifying mechanism, differentiating between individuals who can cognitively and emotionally align with institutional service logic and those who remain excluded due to unresolved normative tensions. By proposing the Stratified Responsiveness Framework (SRF), this study provides a diagnostic tool for identifying these gaps and advancing more ethically attuned, inclusive public service systems.

### Policy implications

Enhancing digital eldercare governance requires a shift beyond infrastructure investment toward ensuring services are culturally and ethically legible within local contexts. Policymakers should prioritize the alignment of service design with prevailing norms of care responsibility. Digital eldercare should be strategically framed as a supportive extension of family care rather than a replacement to reduce the perceived threat to traditional kinship roles.

Furthermore, the implementation process should engage trusted community intermediaries such as relatives, community workers, and local leaders to facilitate the translation of institutional care into familiar ethical terms. Public messaging must move beyond functional utility to emphasize dignity, respect, and continuity with shared values. Ultimately, achieving ethical resonance and moral recognition must be treated as a fundamental prerequisite for service legitimacy, standing alongside usability and accessibility as core pillars of digital welfare effectiveness.

### Limitations and future research

Despite its methodological rigor, this study has several limitations. First, while the structural equation model demonstrated satisfactory fit and stability within Chongqing, these findings are bounded by the regional focus and may reflect the normative homogeneity of the sampled rural areas. Future research should utilize comparative frameworks across diverse geographic settings and service types to test the broader applicability of the Stratified Responsiveness Framework. Second, the cross-sectional nature of the data limits observations of the long-term evolution of ethical lag. As digital welfare infrastructures mature, the negotiation between individual care norms and institutional logic may shift, requiring longitudinal designs to track how moral adaptation unfolds and whether ethical resonance strengthens with prolonged technology exposure. Finally, expanding the inquiry to include diverse regional governance models or urban–rural comparisons will be essential for refining context-sensitive digital inclusion strategies and ensuring the universal legitimacy of digital social welfare systems.

## Supplementary Information

Below is the link to the electronic supplementary material.

Supplementary Material 1. Appendix Table A1. Full Correlation Matrix of All Variables. Appendix Table A2 reports the complete structural equation modeling (SEM) results with control variables, allowing readers to evaluate the stability of the estimated effects when accounting for demographic and contextual factors. Appendix Table A2. Full Structural Model Results with Control Variables. Appendix Table A3 provides robustness checks of the structural model, demonstrating that the main path coefficients remain consistent across alternative specifications. These supplementary tables are supplied in their final form by the authors and will be published as submitted. Appendix Table A3. Robustness Checks of the Structural Model

## Data Availability

The interview data generated and analyzed in this study are not publicly available due to confidentiality restrictions. Anonymized excerpts are available from the corresponding author upon reasonable request.
