# Supplementary material for: Beyond technical access in digital eldercare: how ethical lag shapes stratified responsiveness to institutional welfare in rural China
Source: BMC Geriatr. 2026 Jun 10;26:959. doi: 10.1186/s12877-026-07768-1 (PMC13377828; doi:10.1186/s12877-026-07768-1)
Supplement: Supplementary file 1 — Supplementary Material 1. Appendix Table A1. Full Correlation Matrix of All Variables. Appendix Table A2 reports the complete structural equation modeling (SEM) results with control variables, allowing readers to evaluate the stability of the estimated effects when accounting for demographic and contextual factors. Appendix Table A2. Full Structural Model Results with Control Variables. Appendix Table A3 provides robustness checks of the structural model, demonstrating that the main path coefficients remain consistent across alternative specifications. These supplementary tables are supplied in their final form by the authors and will be published as submitted. Appendix Table A3. Robustness Checks of the Structural Model [file 12877_2026_7768_MOESM1_ESM.docx]

**Appendix Table A1. Full Correlation Matrix of All Variables**

|  | 1 | 2 | 3 | 4 | 5 | 6 | 7 | 8 | 9 | 10 | 11 | 12 |
| --- | --- | --- | --- | --- | --- | --- | --- | --- | --- | --- | --- | --- |
| 1.PDP | 1 |  |  |  |  |  |  |  |  |  |  |  |
| 2.DC | 0.448*** | 1 |  |  |  |  |  |  |  |  |  |  |
| 3.CA | 0.390*** | 0.543*** | 1 |  |  |  |  |  |  |  |  |  |
| 4.PE | 0.350*** | 0.299*** | 0.414*** | 1 |  |  |  |  |  |  |  |  |
| 5.PU | 0.180*** | 0.156** | 0.193*** | 0.583*** | 1 |  |  |  |  |  |  |  |
| 6.BI | 0.367*** | 0.369*** | 0.460*** | 0.654*** | 0.572*** | 1 |  |  |  |  |  |  |
| 7.Gender | -0.01 | 0.006 | 0.014 | 0.016 | 0.019 | -0.038 | 1 |  |  |  |  |  |
| 8.Age | -0.019 | -0.041 | 0.012 | -0.058 | -0.072 | -0.035 | -0.031 | 1 |  |  |  |  |
| 9.Income | 0.038 | -0.036 | -0.01 | 0.015 | 0 | 0.044 | -0.081 | 0.015 | 1 |  |  |  |
| 10.Lifestyle | 0.054 | 0.048 | -0.006 | 0.078 | 0.039 | 0.039 | -0.032 | 0.02 | 0.049 | 1 |  |  |
| 11.Health | -0.052 | 0.041 | 0.048 | 0.016 | 0.001 | -0.024 | -0.026 | -0.065 | 0.021 | -0.003 | 1 |  |
| 12.Proficiency | -0.078 | -0.103* | -0.053 | -0.101* | -0.110* | -0.148** | -0.046 | -0.028 | 0.029 | -0.041 | 0.047 | 1 |

***Note:*** *N = 400. *** p < 0.001, ** p < 0.01, * p < 0.10.*

***Variable abbreviations:*** *PDP= Privacy Data Protection; DC=Dignity in Care; CA=Care Acceptability; PE = Perceived Ease of Use; PU = Perceived Usefulness; BI = Behavioral Intention.*

**Appendix Table A2. Full Structural Model Results with Control Variables**

| **Dependent Variable** | **Predictor Variable** | **β** | **B** | **S.E.** | **t-value** | **p-value** |
| --- | --- | --- | --- | --- | --- | --- |
| **Perceived Ease of Use (PE)** | | | | | | |
|  | ***(Main Predictors)*** |  |  |  |  |  |
|  | PDP | 0.254 | 0.321 | 0.114 | 2.811 | 0.005** |
|  | DC | -0.154 | -0.194 | 0.129 | -1.509 | 0.131 |
|  | CA | 0.487 | 0.614 | 0.135 | 4.567 | 0.000*** |
|  | ***(Control Variables)*** |  |  |  |  |  |
|  | Gender (Female) | 0.019 | 0.048 | 0.129 | 0.374 | 0.708 |
|  | Age (70-79) | -0.091 | -0.244 | 0.170 | -1.437 | 0.151 |
|  | Age (≥ 80) | -0.089 | -0.231 | 0.156 | -1.477 | 0.140 |
|  | Income (Child support) | 0.065 | 0.231 | 0.190 | 1.216 | 0.224 |
|  | Income (Post-retirement work) | -0.011 | -0.038 | 0.181 | -0.210 | 0.834 |
|  | Income (Financial management, etc.) | -0.015 | -0.061 | 0.231 | -0.263 | 0.793 |
|  | Income (Personal savings) | -0.022 | -0.090 | 0.205 | -0.440 | 0.660 |
|  | Income (Other) | 0.075 | 0.445 | 0.242 | 1.842 | 0.065* |
|  | Lifestyle (Institutional care) | 0.090 | 0.230 | 0.133 | 1.733 | 0.083* |
|  | Lifestyle (Other) | -0.054 | -0.963 | 0.625 | -1.541 | 0.123 |
|  | Health status (Occasional discomfort) | -0.091 | -0.255 | 0.175 | -1.458 | 0.145 |
|  | Health status (Fair health, needs help) | -0.020 | -0.065 | 0.197 | -0.332 | 0.740 |
|  | Health status (Not very healthy) | 0.027 | 0.093 | 0.212 | 0.441 | 0.659 |
|  | Health status (Requires long-term care) | -0.033 | -0.124 | 0.226 | -0.549 | 0.583 |
|  | Proficiency (Basic functions only) | -0.047 | -0.170 | 0.198 | -0.859 | 0.391 |
|  | Proficiency (Can use common functions) | -0.193 | -0.538 | 0.175 | -3.079 | 0.002** |
|  | Proficiency (Can use most functions) | -0.080 | -0.266 | 0.196 | -1.356 | 0.175 |
|  | Proficiency (Proficient in using) | -0.053 | -0.212 | 0.238 | -0.891 | 0.373 |
| **Perceived Usefulness (PU)** | | | | | | |
|  | ***(Main Predictors)*** |  |  |  |  |  |
|  | PE | 0.679 | 0.756 | 0.087 | 8.726 | 0.000*** |
|  | ***(Control Variables)*** |  |  |  |  |  |
|  | Gender (Female) | -0.001 | -0.004 | 0.121 | -0.035 | 0.972 |
|  | Age (70-79) | -0.011 | -0.033 | 0.165 | -0.200 | 0.842 |
|  | Age (≥ 80) | -0.050 | -0.144 | 0.149 | -0.970 | 0.332 |
|  | Income (Child support) | -0.059 | -0.233 | 0.181 | -1.285 | 0.199 |
|  | Income (Post-retirement work) | 0.056 | 0.220 | 0.200 | 1.103 | 0.270 |
|  | Income (Financial management, etc.) | -0.040 | -0.181 | 0.186 | -0.970 | 0.332 |
|  | Income (Personal savings) | 0.058 | 0.263 | 0.221 | 1.191 | 0.234 |
|  | Income (Other) | -0.086 | -0.572 | 0.324 | -1.761 | 0.078* |
|  | Lifestyle (Institutional care) | -0.014 | -0.039 | 0.123 | -0.315 | 0.753 |
|  | Lifestyle (Other) | 0.015 | 0.291 | 0.601 | 0.485 | 0.628 |
|  | Health status (Occasional discomfort) | -0.078 | -0.244 | 0.165 | -1.478 | 0.140 |
|  | Health status (Fair health, needs help) | -0.076 | -0.283 | 0.189 | -1.495 | 0.135 |
|  | Health status (Not very healthy) | -0.101 | -0.387 | 0.213 | -1.821 | 0.069* |
|  | Health status (Requires long-term care) | -0.019 | -0.080 | 0.210 | -0.382 | 0.702 |
|  | Proficiency (Basic functions only) | -0.030 | -0.121 | 0.197 | -0.615 | 0.538 |
|  | Proficiency (Can use common functions) | -0.079 | -0.245 | 0.160 | -1.534 | 0.125 |
|  | Proficiency (Can use most functions) | -0.094 | -0.345 | 0.182 | -1.90 | 0.057* |
|  | Proficiency (Proficient in using) | 0.013 | 0.056 | 0.244 | 0.229 | 0.819 |
| **Behavioral Intention (BI)** | | | | | | |
|  | ***(Main Predictors)*** |  |  |  |  |  |
|  | PDP | 0.090 | 0.234 | 0.181 | 1.293 | 0.196 |
|  | DC | 0.008 | 0.022 | 0.197 | 0.112 | 0.911 |
|  | CA | 0.230 | 0.601 | 0.247 | 2.430 | 0.015* |
|  | PE | 0.524 | 1.084 | 0.292 | 3.705 | 0.000*** |
|  | PU | 0.244 | 0.453 | 0.144 | 3.147 | 0.002** |
|  | ***(Control Variables)*** |  |  |  |  |  |
|  | Gender (Female) | -0.075 | -0.398 | 0.221 | -1.799 | 0.072* |
|  | Age (70-79) | -0.002 | -0.011 | 0.275 | -0.038 | 0.969 |
|  | Age (≥ 80) | 0.020 | 0.106 | 0.257 | 0.414 | 0.679 |
|  | Income (Child support) | 0.065 | 0.475 | 0.329 | 1.444 | 0.149 |
|  | Income (Post-retirement work) | 0.089 | 0.654 | 0.328 | 1.994 | 0.046* |
|  | Income (Financial management, etc.) | 0.037 | 0.310 | 0.366 | 0.846 | 0.397 |
|  | Income (Personal savings) | 0.051 | 0.427 | 0.394 | 1.085 | 0.278 |
|  | Income (Other) | -0.023 | -0.288 | 0.414 | -0.695 | 0.487 |
|  | Lifestyle (Institutional care) | -0.032 | -0.168 | 0.220 | -0.766 | 0.444 |
|  | Lifestyle (Other) | 0.009 | 0.320 | 1.125 | 0.285 | 0.776 |
|  | Health status (Occasional discomfort) | -0.036 | -0.211 | 0.295 | -0.715 | 0.474 |
|  | Health status (Fair health, needs help) | 0.005 | 0.036 | 0.322 | 0.112 | 0.911 |
|  | Health status (Not very healthy) | -0.081 | -0.573 | 0.358 | -1.602 | 0.109 |
|  | Health status (Requires long-term care) | -0.032 | -0.247 | 0.342 | -0.721 | 0.471 |
|  | Proficiency (Basic functions only) | -0.074 | -0.555 | 0.338 | -1.643 | 0.100 |
|  | Proficiency (Can use common functions) | -0.089 | -0.514 | 0.291 | -1.767 | 0.077* |
|  | Proficiency (Can use most functions) | -0.031 | -0.211 | 0.324 | -0.651 | 0.515 |
|  | Proficiency (Proficient in using) | -0.079 | -0.652 | 0.364 | -1.790 | 0.073* |

***Note:*** *β represents standardized coefficients; B represents unstandardized coefficients. This table reports the full structural model including all control variables. The reference categories are: Gender (Male); Age (60-69); Income (National insurance); Lifestyle (Home-based care); Health status (Very healthy); Digital proficiency (Cannot use at all). *** p < 0.001, ** p < 0.01, * p < 0.10.*

**Appendix Table A3. Robustness Checks of the Structural Model**

| **Model** | **PDP→BI** | **DC→BI** | **CA→BI** | **PE→BI** | **PU→BI** | **CFI** | **TLI** | **RMSEA** | **SRMR** |
| --- | --- | --- | --- | --- | --- | --- | --- | --- | --- |
| Model 1 (Baseline Model) | 0.054 | 0.042 | 0.18 | 0.599 | 0.206 | 0.994 | 0.991 | 0.016 | 0.028 |
| Model 2 (Remove PDP→PU Path) | 0.071 | 0.034 | 0.183 | 0.585 | 0.215 | 0.993 | 0.991 | 0.016 | 0.028 |
| Model 3 (Remove DC→PU Path) | 0.06 | 0.027 | 0.191 | 0.595 | 0.207 | 0.994 | 0.991 | 0.016 | 0.028 |
| Model 4 (Remove CA→PU Path) | 0.055 | 0.017 | 0.229 | 0.565 | 0.223 | 0.991 | 0.988 | 0.019 | 0.029 |
| Model 5 (Add PE↔PU Covariance) | 0.054 | 0.042 | 0.18 | 0.599 | 0.206 | 0.994 | 0.991 | 0.016 | 0.028 |

***Note:*** *Results show that the main path coefficients remain stable across alternative model specifications, supporting the robustness of the findings. Abbreviations: PDP= Privacy Data Protection; DC=Dignity in Care; CA=Care Acceptability; PE = Perceived Ease of Use; PU = Perceived Usefulness; BI = Behavioral Intention; CFI = Comparative Fit Index; TLI = Tucker-Lewis Index; RMSEA = Root Mean Square Error of Approximation; SRMR = Standardized Root Mean Square Residual.*
